# *Zea mays*–Derived Zinc Oxide Nanoparticles Exhibiting Enhanced Antioxidant, Antibacterial, and Wound‐Healing Activities

**DOI:** 10.1155/bmri/2670207

**Published:** 2026-02-04

**Authors:** Aqsa Khalid, Raheela Waheed, Zermina Rashid, Farah Deeba, Ambreen Aleem, Mohamed Deifallah Yousif

**Affiliations:** ^1^ Department of Biochemistry and Biotechnology, The Women University Multan, Multan, Pakistan, wum.edu.pk; ^2^ Department of Biosciences and Technology, Emerson University Multan, Multan, Pakistan; ^3^ Department of Pharmacy, The Women University Multan, Multan, Pakistan, wum.edu.pk; ^4^ Faculty of Pharmacy, Bahauddin Zakariya University, Multan, Pakistan, bzu.edu.pk; ^5^ UCL School of Pharmacy, University College London, London, UK, ucl.ac.uk

**Keywords:** antibacterial, antioxidant, topical gel, wound healing, *Zea mays*, ZnO nanoparticles

## Abstract

Infections are a cause of delayed wound healing, and the development of effective therapeutic strategies remains a key challenge. This study is aimed at developing and evaluating *Zea mays* leaf extract‐mediated zinc oxide nanoparticles (ZnZM NPs) for their antibacterial, antioxidant, and wound‐healing potential. *Z*. *mays* leaf extract was utilized for the green synthesis of ZnZM NPs, which were characterized using multiple analytical techniques. The UV‐visible spectrum exhibited a characteristic sharp absorption peak at 390 nm, and energy‐dispersive X‐ray (EDX) spectrometry confirmed the presence of zinc and oxygen. FT‐IR confirmed that the phytochemicals from *Z*. *mays* extract were involved in the reduction and capping of NPs. The ZnZM NPs were slightly aggregated, partially spherical, and crystalline, with an average crystallite size of 10.86 nm. The nanoparticles exhibited significant antibacterial activity against *E. coli*, *P. aeruginosa*, and *K. pneumoniae*. They also exhibited notable ferric ion‐reducing power and free radical‐scavenging ability. Topical gels containing 1% ZnZM NPs accelerated wound healing in rats compared with the control and standard (commercial product). Histopathological studies further confirmed enhanced tissue regeneration and accelerated wound healing in rats treated with NPs compared with the control and standard groups. Our findings suggest that biosynthesized zinc oxide nanoparticles possess antibacterial, antioxidant, and accelerated wound‐healing properties and can serve as an economic, safe, and sustainable nanomedicine for use in clinical settings.

## 1. Introduction

Nanoparticles are submicron‐sized particles that can be made from organic or inorganic materials. These miniaturized materials have various properties that allow their use in different industries, including engineering, medicine, manufacturing, and environmental fields [1]. They can be synthesized chemically, mechanically, or through natural processes. The chemical and mechanical processes involved in their synthesis are associated with numerous problems, such as complexity, cost, and the use of harmful solvents.

Green synthesis of NPs, which involves the use of natural products such as plant extracts, is mainly concerned with avoiding the harmful chemicals utilized in the chemical synthesis [2, 3]. Biologically generated metallic nanoparticles are useful in various applications, such as protection against infectious microorganisms, drug transportation, cancer treatment, and sensor construction, owing to their distinctive characteristics [4–8]. Their utility also extends to therapeutic areas, such as wound healing and antioxidant activity [9, 10]. ZnO (zinc oxide) nanoparticles, among metallic nanoparticles, have received more attention than the others due to their inert nature, minimal rusting features, and economic effectiveness. ZnO nanoparticles possess a wide range of therapeutic applications, including anticancer, antimicrobial, and anti‐inflammatory treatments, as well as wound healing and drug delivery. Zinc (Zn) is an essential trace element in the human physiological system. Owing to its low toxicity and high biodegradability, which is attributed to the solubility of Zn^2+^ ions, ZnO exhibits excellent biocompatibility, making it suitable for various biomedical applications [11, 12].

The creation of ZnO nanoparticles from plants is a simple, inexpensive, and sustainable procedure [5]. Macromolecules and compounds found in plants, such as flavonoids, coenzyme‐based intermediates, phenols, vitamins, and carbohydrates, are normally involved in the process of NPs′ formation. The hydroxyl, carbonyl, and amine functional groups in plant metabolites react with metal ions to shrink them into nanoscale particles [6]. *Zea mays* L., usually referred to as corn or maize, is an important cereal crop around the world. It contains nutrients and numerous significant phytochemicals, such as phytosterols, carotenoids, flavonoids, saponins, alkaloids, glycosides, and phenolic compounds. Traditionally, in some countries, it has been used to treat diabetes, and its extract is known to have anti‐inflammatory effects [13].

This study describes the biosynthesis of ZnO nanoparticles using *Z*. *mays* L. leaf extract. *Z. mays*‐mediated ZnO nanoparticles (ZnZM NPs) were characterized for their physicochemical properties using UV‐vis spectroscopy, scanning electron microscopy (SEM), energy dispersive X‐ray (EDX), FTIR, and X‐ray diffraction (XRD). Their in vitro antibacterial activity against *Escherichia coli*, *Pseudomonas aeruginosa*, and *Klebsiella pneumoniae* and antioxidant activity were also investigated. The optimized ZnZM NPs were incorporated into a carbopol gel to evaluate their potential wound‐healing efficacy.

## 2. Materials and Methods

### 2.1. Materials

Fresh *Z. mays* leaves were gathered from the botanical garden of the Bahauddin Zakariya University (BZU), Multan, Pakistan. Carbopol 934 was purchased from Avon Pharmo Chem (Sri Lanka). Sodium hydroxide and Zn acetate were purchased from Merck (Pakistan). The bacterial strains were provided by the Department of Microbiology, Agriculture University Faisalabad, Pakistan. All chemicals and reagents used in this study were of analytical grade.

### 2.2. Biosynthesis of ZnO Nanoparticles Using *Z*. *mays* L. Leaf Extract

Freshly collected *Z*. *mays* L. leaves were initially washed with tap water, followed by deionized water to remove dust, debris, and surface contaminants. The washed leaves were cut into small pieces, triturated, and subsequently boiled (5 g in 100 mL) in distilled water for half an hour using a water bath to extract the bioactive phytochemicals responsible for metal ion reduction and stabilization. The extract was allowed to cool to room temperature and subsequently filtered through Whatman No. 1 filter paper to remove plant residues. The filtrate was further diluted and used as a bioreductant for the synthesis of zinc oxide nanoparticles (ZnZM NPs) in several optimized batches, as presented in Table 1.

**Table 1:** Dilutions of *Zea mays* leaf extract.

**Batch code**	**Extract volume (mL)**	**DI water volume (mL)**	**Extract concentration (%)**
ZnZM‐1	10	0	5
ZnZM‐2	8	2	4
ZnZM‐3	6	4	3
ZnZM‐4	4	6	2
ZnZM‐5	2	8	1

Abbreviation: DI, deionized.

For the biosynthesis of each batch of ZnZM NPs, typically, 20 mL of prepared *Z*. *mays* L. leaf extract and 80 mL of 0.1‐mM Zn acetate solution (2.159 g/100 mL) were mixed under continuous stirring. The pH of the reaction mixture was maintained at pH 7 using NaOH solution dropwise. The reaction mixture was heated at 70°C with continuous stirring for approximately 1 h. Upon heating, a visible color change to milky white was observed, indicating the reduction of Zn^2+^ ions to ZnO nanoparticles and the onset of particle nucleation. After the reaction was complete, the mixture was left undisturbed at room temperature for 24 h to ensure the complete formation and growth of nanoparticles. The obtained milky colloidal suspension was centrifuged at 9000 rpm for 20 min to remove unreacted precursors. The supernatant was discarded, and the precipitates were washed thrice with distilled water to remove any unreacted precursors and loosely bound phytochemicals. The purified precipitates were then dried at 150°C for 2 h in a hot air oven to obtain fine ZnO nanopowder, which was collected and stored in an airtight container for further physicochemical and biological characterization [5].

### 2.3. Characterization of ZnZM Nanoparticles

#### 2.3.1. UV‐Visible Spectroscopy

The optical properties of the biosynthesized nanoparticles were evaluated using a UV‐visible spectrophotometer (PerkinElmer, Japan). The absorbance spectrum of the colloidal suspension was recorded in the range of 200–800 nm.

#### 2.3.2. ATR FTIR Analysis

The surface chemistry and functional moieties involved in nanoparticle formation were recorded using ATR FT‐IR (Bruker IR, Japan). Dried ZnZM NPs were directly placed on the diamond crystal of the ATR accessory, and spectra were recorded in the range of 4000–400 cm^−1^ at a resolution of 4 cm^−1^.

#### 2.3.3. XRD Analysis

The crystalline nature and phase purity of the synthesized ZnZM NPs were analyzed using XRD spectroscopy (X‐ray diffractometer, JDX 3532; JEOL, Japan). The dried nanoparticle powder was scanned over a 2*θ* range of 10°–80° at a scanning rate of 0.02°/s using Cu K*α* radiation ($\lambda =1.5406\;\overset{{}^{\circ}}{A}$) operated at 40 kV and 30 mA. The average crystallite size was calculated using the Debye–Scherrer equation [14]:

D=Kλβcosθ

where *K* is the shape factor (0.9), *λ* is the X‐ray wavelength, *β* is the full width at half maximum (FWHM), and *θ* is the Bragg angle.

#### 2.3.4. SEM

The surface morphology of the ZnZM NPs was examined using SEM (Philip model CM 200). The dried nanoparticle powder was evenly spread on a carbon‐coated stub and sputter‐coated with a thin layer of gold to improve electrical conductivity. Images were captured at various magnifications [15].

#### 2.3.5. EDX Spectroscopy

Elemental composition and purity of the synthesized nanoparticles were evaluated using EDX spectroscopy coupled with SEM (Model; Inca 200 Oxford, United Kingdom) [16].

### 2.4. Antibacterial Activity

The antibacterial activity of ZnZM NPs against selected bacterial strains was tested using the well diffusion method. Stock cultures of *E*. *coli*, *P*. *aeruginosa*, and *K*. *pneumoniae* were cultivated in nutrient broth, and the cultures were adjusted to 0.5 McFarland standard and uniformly spread on Mueller Hinton Agar (MHA) plates. Wells (8 mm) were created, 50 *μ*L of ZnZM NPs were added into the wells and incubated at 37°C for 24 h. *Z. mays* leaf extract and distilled water were used as negative controls, whereas ciprofloxacin (5 *μ*g) and amikacin (30 *μ*g) were used as positive controls. After the 24 h, zones of inhibition (ZOI) were recorded in millimeters [17].

### 2.5. Antioxidant Assay

The antioxidant activity of the synthesized ZnO nanoparticles was determined using the 1,1‐diphenyl‐2‐picrylhyrazyl (DPPH) test [18] and ferric ion (Fe^3+^)‐reducing power assay [19]. Ascorbic acid was used as the reference standard.

#### 2.5.1. DPPH Radical‐Scavenging Activity

Five different concentrations of ZnO nanoparticle batch ZnZM‐1 were prepared (500, 250, 150, 62, and 31 *μ*g/mL). A total of 100 *μ*L of nanoparticle solution was added to 900 *μ*L of DPPH solution from stock solution and incubated (37°C) for 15–20 min. The absorbance was recorded at 517 nm in triplicate [11], and the DDPH free radical scavenging (%) was estimated by using the following formula:

%scavenging=A0−AzA0×100

where A_0_ is absorbance of the control and Az is the absorbance of the test sample.

#### 2.5.2. Fe^3+^‐Reducing Power Assay

In this approach, 1 mL of ZnO nanoparticles at various concentrations (31, 62, 125, 250, and 500 mg/mL) was combined with 2.5 mL of phosphate buffer (0.2 M, *p* *H* = 6.6). The mixtures were then treated with 2.5 mL of 1% (w/v) potassium ferricyanide and maintained at 50°C for 20 min. After incubation, a 2.5‐mL trichloroacetic acid solution (10%, w/v) was added to stop the reaction. The solution was centrifuged (3000 rpm, 3 min), the supernatant was diluted with an equal volume of distilled water, and treated with a 500‐*μ*L FeCl_3_ solution (0.1%, w/v) for 30 min. The solution developed a blue color, and the change in absorbance was measured at 700 nm using a spectrophotometer. All reactions were performed in triplicate.

### 2.6. Preparation and Evaluation of ZnZM NPs–Loaded Topical Gel

ZnO nanoparticles from batch ZnZM‐1 (1%) were dispersed in 20‐mL distilled water. Carbopol 934 (0.1 g) was added to the ZnZM NP dispersion and stirred continuously for 1 h. After complete hydration of carbopol, the dispersion was neutralized by adding 2–3 drops of triethanolamine, which resulted in the formation of a gel. Then, 2% (w/w) glycerol was used as a humectant. The physicochemical properties of the synthesized gel (Figure S1), including color, odor, consistency, homogeneity, and pH, were evaluated [20].

### 2.7. Skin Irritation Test

Skin irritation was assessed in healthy Wistar rats to determine the sensitivity of rat skin to the NP‐loaded topical gel. For this purpose, nanoparticle‐loaded gels were applied to the back of the shaved rats (*n* = 6). Erythema and edema were evaluated at 6, 12, 24, and 48 h [21].

### 2.8. In Vivo Wound‐Healing Evaluation

The animals were obtained from the Faculty of Pharmacy Animal House at BZU, Multan, Pakistan. All procedures were approved by the Women′s University Multan Ethical Committee for the Use of Laboratory Animals. Eighteen healthy adult Wistar rats (~150–160 g) were kept in a conventional laboratory setting on a 12‐h day and 12‐h night cycle with free access to standard food and water. For in vivo wound‐healing studies, the animals were anesthetized using lignocaine gel, and the ventral skin was shaved, disinfected with ethanol (70%), and randomly divided into three groups, with each group containing six rats. Group 1 rats were used as the controls (untreated). Group 2 rats were used as the standard and treated with a commercial product (quench cream). Group 3 rats were treated with ZnZM NP (1%)–loaded carbopol gel. Wound‐healing progression and dressing were monitored for these rats on the 3rd, 5th, 7th, and 9th days. The percentage reduction in wound size was estimated using the following formula:

%wound contraction=w0−wdw0×100

where w_0_ is the size of the wound on Day 0 and w_d_ is the size of the wound on days of measurement (i.e., 3rd, 5th, 7th, and 9th days) [20].

### 2.9. Histopathological Studies

For histopathological evaluation, the rats were euthanized on Day 10, and a portion of the regenerated tissue was excised. The collected samples were sectioned using a microtome, fixed in 10% formalin, and stained with hematoxylin and eosin (H and E). The stained samples were subsequently examined under optical microscopy.

### 2.10. Statistical Analysis

All data are presented as mean ± standard deviation. The differences in percentage wound healing between the groups were statistically compared using one‐way ANOVA, followed by the least significant difference (LSD) test for intergroup comparisons using SPSS software (SPSS Inc. Chicago, Illinois United States). Statistical significance was set at *p* ≤ 0.05 [20].

## 3. Results and Discussion

### 3.1. Physicochemical Characterization

#### 3.1.1. UV‐Vis Spectroscopy Analysis

UV‐vis spectroscopy is an excellent method for validating and characterizing the surface plasmon resonance (SPR)‐based synthesis of NPs. The SPR in the UV‐vis portion of the spectrum is created by resonant collective oscillations of conduction electrons in the transverse direction of the electromagnetic field [6]. All five batches of ZnZM NPs showed an absorption peak at approximately 390 nm (Figure 1a). The observed SPR peak is indicative of the characteristic band gap of ZnO, reflecting the electron transition from the valence to the conduction band [12]. The maximum absorption was recorded at 395 nm for ZnZM NPs synthesized with the most concentrated plant extract, which is high in phytochemical content, resulting in an increase in NPs production and an improvement in the absorbance value. In a similar study, ZnO nanoparticles synthesized using a higher concentration of *C. pedata* extract showed more absorption in comparison with ZnO NPs synthesized from lower concentrations [22].

Figure 1Physicochemical characteristics of the ZM plant extract and ZnZM NPs. (a) Color change after the formation of ZnZM NPs. (b) SEM images of ZnZM NPs. (c) XRD of ZnZM NPs. (d) FT‐IR spectra of the ZM plant extract. (e) FT‐IR spectra of ZnZM NPs.

**Figure:**
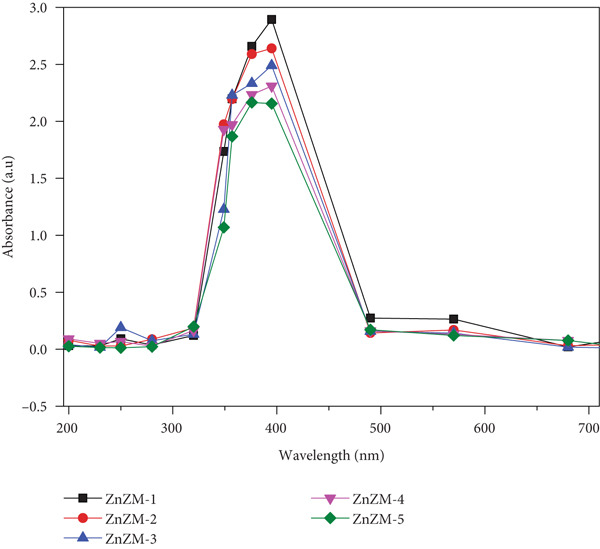
(a)

**Figure:**
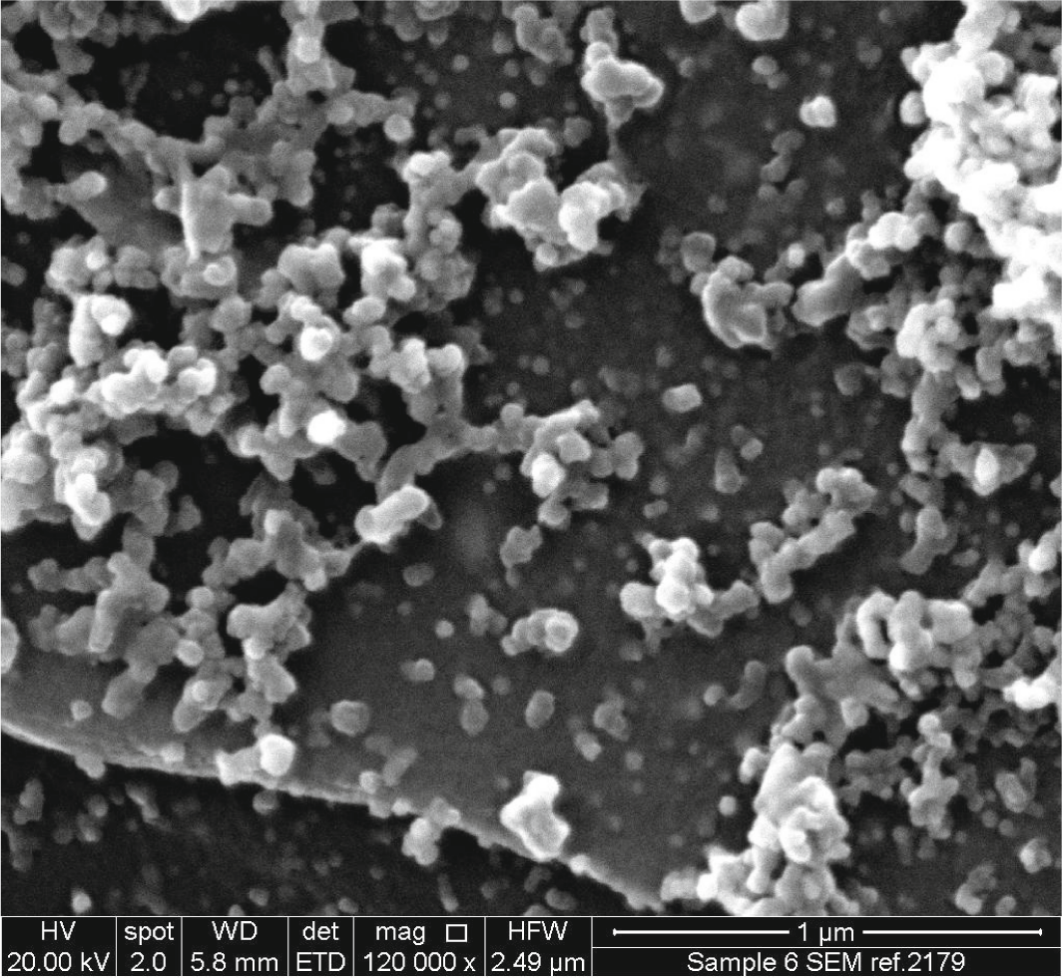
(b)

**Figure:**
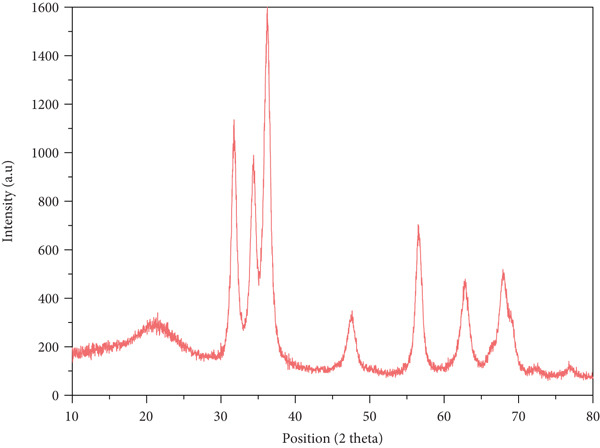
(c)

**Figure:**
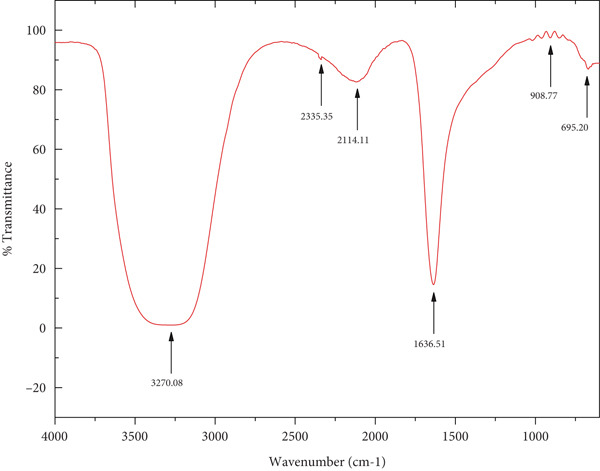
(d)

**Figure:**
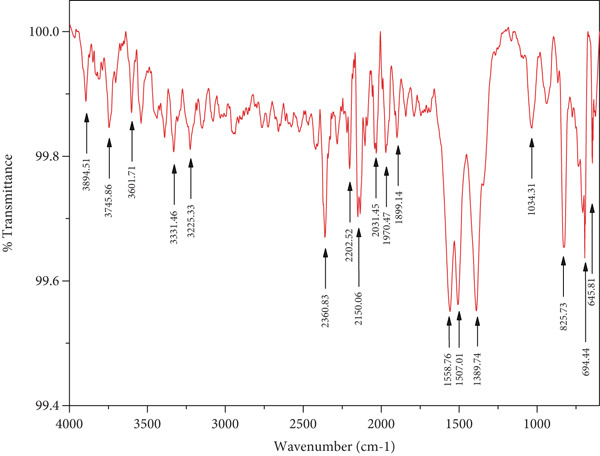
(e)

#### 3.1.2. SEM Analysis

SEM confirmed the crystalline assembly of ZnZM NPs (Figure 1b). The SEM image shows that the ZnZM NPs formed a large network system by interlinking with one another. These interlinked particles were micron‐sized. The micrograph indicates that the ZnZM NPs were spherical or semispherical in shape [5].

#### 3.1.3. XRD Analysis

The crystal alignment, phase composition, and phase identification of the biologically produced ZnZM NPs were determined using X‐ray crystallography. The X‐ray diffractogram of ZnZM NPs (Figure 1c) exhibited distinct diffraction peaks at the 2*φ* values of 31.78, 34.4, 36.34, 47.72, 56.7, 63.02, and 68.08 [22], which were indexed to (100, 002, 101, 102, 110, 103, and 200) crystallographic planes, respectively. These diffraction features are due to the constructive interference of X‐rays scattered by atomic planes within the crystal lattice. The observed reflections were in good agreement with JCPDS card No. 01‐080‐0075, confirming that ZnO has a hexagonal wurtzite crystal structure. In this agreement, the lattice is composed of alternating layers of Zn and oxygen (O) atoms, and the assigned (hkl) indices represent the specific crystallographic orientations of the planes within the lattice. The experimental results were validated using previously reported diffraction patterns of ZnO nanoparticles [23]. The average crystallite size was estimated to be 10.86 nm using the Scherrer equation [14].

#### 3.1.4. FT‐IR Analysis

The different physiologically active biomolecules in the plant extracts that were actively involved in the formation of ZnO nanoparticles were identified using FT‐IR spectroscopy. Figure 1d,e shows the FT‐IR spectra of the leaf extracts and ZnZM NPs, respectively. The FT‐IR spectrum of *Z*. *mays* leaf extract displayed prominent peaks at 3270.08, 2335.35, 2114.11, 1636.51, 908.77, and 695.20 cm^−1^. The broad band at 3270.08 cm^−1^ corresponds to the O–H stretching vibrations of alcoholic and phenolic groups, whereas those at 2335.35 and 2114.11 cm^−1^ are attributed to *C* ≡ *O* and *C* ≡ *C* stretching, respectively. The absorption at 1636.51 cm^−1^ indicates the presence of *C* = *O* (carbonyl group), whereas bands at 908.77 and 695.20 cm^−1^ are characteristic of C–Cl stretching. These functional groups suggest the involvement of polyphenols, proteins, and other phytochemicals from the extract in the reduction and stabilization of the nanoparticles.

In the FT‐IR spectra of ZnZM NPs, broad absorption bands at 3894.51, 3745.86, and 3601.70 cm^−1^ were observed, reflecting hydrogen‐bonded hydroxyl groups on the nanoparticle surface. Peaks observed at 3331.46 and 3225.33 cm^−1^ were associated with the O–H and N–H stretching of amines and alcohols. Absorption at 2360.83, 2202.52, 2150.06, and 2031.45 cm^−1^ were assigned to *C* ≡ *N* and *C* ≡ *C* stretching, confirming contributions from nitriles and alkynes. Bands in the region of 2000–1500 cm^−1^ were attributed to *C* = *O* stretching of the carbonyl group, whereas those in the range of 1500–1000 cm^−1^ represented C–N stretching and C–H bending for amines and alkanes, respectively. These shifts and newly formed bands confirmed the capping and stabilization of ZnZM NPs by biomolecules from the *Z*. *mays* leaf extract [24].

#### 3.1.5. EDX Analysis

**Figure 2:**
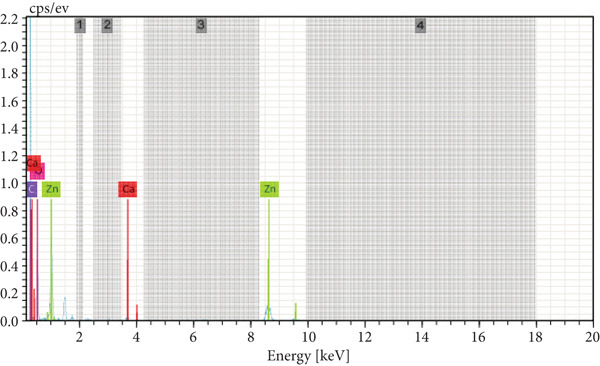
EDX spectrum of synthesized ZnO nanoparticles.

The elemental composition, structural configuration, and chemical constituents of ZnZM NPs were investigated using EDX spectroscopy (Figure 2). The EDX spectrum showed strong signals corresponding to Zn and O, confirming them as the predominant constituents of the biosynthesized ZnZM NPs, with no detectable impurities. Minor signals of carbon and calcium were also observed, which may be attributed to plant‐derived phytochemicals involved in the reduction and stabilization of the nanoparticles or to the residues from the supporting substrate used during SEM analysis, which is consistent with the previous work on the synthesis of ZnO NPs reported by Pammi et al. [23].

### 3.2. Antibacterial Activity

**Figure 3:**
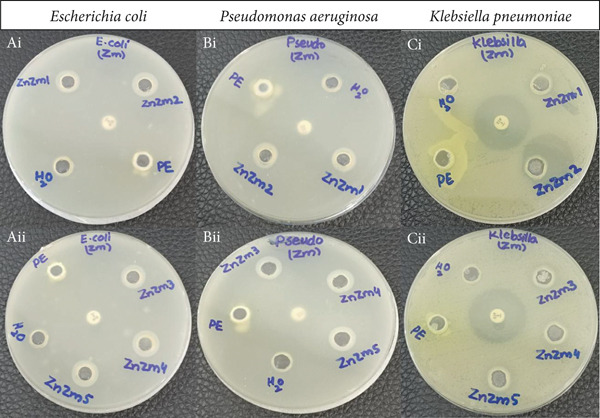
Antibacterial activity of ZnZM NPs against *E. coli*, *P. aeruginosa*, and *K. pneumoniae*.

ZnO nanoparticles have a higher surface area and propensity to block various pathogens and release antibacterial substances when they come into contact with bacterial cell walls. The mechanism by which ZnO nanoparticles kill bacteria is by disrupting the integrity of the membrane and damaging the phospholipid bilayer [25]. In Figure 3, our findings showed that all the investigated bacterial strains were significantly inhibited by the biosynthesized ZnZM NPs, with the maximum activity against *E. coli* (Figure 3 Ai and Aii), then *P. aeruginosa* (Figure 3 Bi and Bii), and the lowest activity against *K. pneumoniae* (Figure 3 Ci and Cii). The positive control ciprofloxacin disk exhibited antibacterial activity (ZOI = 28 mm) against *K. pneumoniae* but not against *E. coli* or *P. aeruginosa* (Table 2). However, all three tested strains were resistant to amikacin (positive control). The effects of the solvent (water) and plant extract on the growth of bacterial strains were tested and used as negative controls. Our investigation showed that no ZOIs were observed for the solvent or plant extracts, suggesting that they do not contribute to the antibacterial activity of ZnZM NPs. The ZnZM NPs demonstrated ZOIs of 11–14 mm against *E. coli*, 11–13 mm against *P. aeruginosa*, and 10–13 mm against *K. pneumoniae*. Notably, increasing the concentration of the plant extract during ZnO NP synthesis enhanced the antibacterial efficacy of ZnZM NPs, which is in agreement with previous reports [26].

**Table 2:** Diameter of the zone of inhibition (ZOI) against different bacterial strains.

**Sample**	**Zone of inhibition (mm ± SD)**		
	***E. coli***	***P. aeruginosa***	***K. pneumoniae***
ZnZM5	14 ± 1.2	12.66 ± 0.57	12.66 ± 0.57
ZnZM4	12.66 ± 0.57	12.33 ± 0.59	12.33 ± 1.52
ZnZM3	12 ± 1.15	12 ± 1.1	12.33 ± 0.57
ZnZM2	11 ± 0.56	11 ± 1.3	10.66 ± 0.74
ZnZM1	10.33 ± 0.57	10.33 ± 0.56	10.33 ± 0.52
PE	0	0	0
Ciprofloxacin	0	0	28 ± 0.59
Amikacin	0	0	0
Water	0	0	0

Abbreviation: PE, plant extract.

### 3.3. Antioxidant Activity

The antioxidant properties of the synthesized ZnO nanoparticles (ZnZM NPs) were assessed using DPPH and FRAP assays, with ascorbic acid serving as the reference standard. ZnO nanoparticles synthesized using plant extracts demonstrated significant radical‐scavenging activity. In this study, varying concentrations of ZnZM NPs were prepared and combined with DPPH solution, followed by incubation in the dark. The resulting color change from purple to yellow was measured spectrophotometrically at 517 nm, with ascorbic acid employed as a positive control (Figure 4). The results indicated a concentration‐dependent increase in radical‐scavenging activity, consistent with previous reports [27]. Specifically, the radical‐scavenging activity of ZnZM NPs at concentrations ranging from 31 to 500 *μ*g/mL varied from 44.13% to 95.37%, whereas ascorbic acid exhibited activity between 46.13% and 97.96% (Figure 4a).

Figure 4(a) DPPH radical‐scavenging activity of ZnZM NPs and ascorbic acid (standard) at different concentrations (*n* = 3, mean ± SD). (b) Ferric ion‐reducing activity of ZnZM NPs and ascorbic acid (standard) at different concentrations (*n* = 3, mean ± SD).

**Figure:**
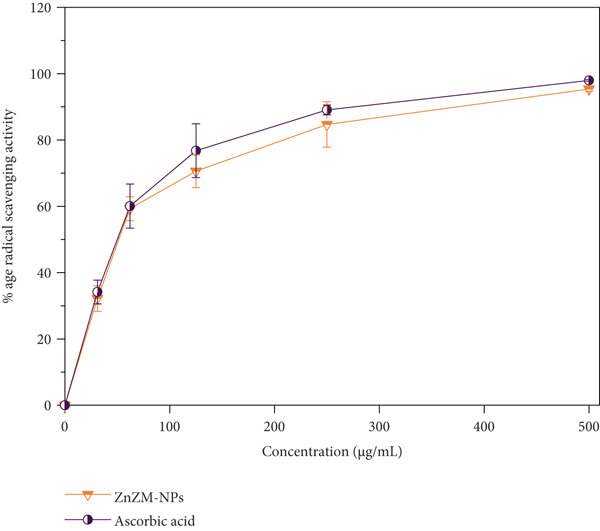
(a)

**Figure:**
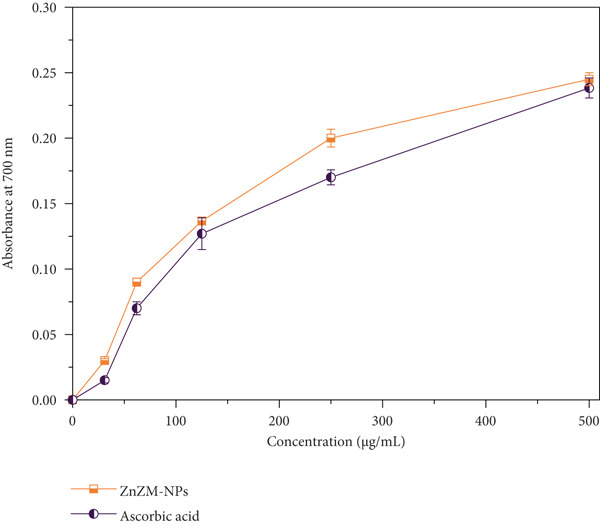
(b)

The FRAP assay evaluates the reducing power of NPs by measuring its ability to reduce Fe^3+^ to ferrous ions (Fe^2+^), forming a blue ferrous–tripyridyl triazine complex. The data revealed that the ferric‐reducing power of ZnZM NPs increased with increasing nanoparticle concentration (Figure 4b), similar to the results reported previously [28].

### 3.4. In Vivo Wound Healing

Open and untreated wounds are vulnerable to bacterial infections. ZnO NPs have a broad‐spectrum antibacterial activity and can modulate inflammation by regulating cytokine production, reducing the release of inflammatory cytokines and growth factors, and promoting wound healing and scar formation [29]. Accordingly, a 1% ZnZM NP–loaded carbopol gel was prepared (Figure S1) and its wound contraction efficacy was assessed in rats, with results compared with those of a control group and a standard commercial product (Figure 5a,b). Wound diameters were measured on Days 3, 5, 7, and 9 (Figure 5a) for quantitative analysis of wound healing (Figure 5a). In the control group (Group 1), wound contraction was 12.75*%* ± 2.46, 24.60*%* ± 2.39, 34.22*%* ± 2.84, and 44.55*%* ± 2.94 on Days 3, 5, 7, and 9, respectively. The standard treatment group (Group 2) showed wound contraction rates of 20.06 ± 6.66, 44.60 ± 4.10, 53.07 ± 3.43, and 62.50 ± 4.10 over the same time period. Rats treated with ZnZM NP–loaded topical gel (Group 3) showed the highest wound contraction of 22.42 ± 14.54, 49.48 ± 2.92, 58.62 ± 2.68, and 69.00 ± 1.66 on Days 3, 5, 7, and 9, respectively, demonstrating superior wound healing in comparison with both control and standard groups [30]. In a similar study, *Lawsonia inermis* leaf extract‐mediated ZnO NPs exhibited increased wound contraction and reduced healing time compared with the control group. Similarly, accelerated wound contraction was observed in another study, in which ZnO NPs were biosynthesized using *Bauhinia variegata* leaf extract [31].

Figure 5(a) Wound healing in rats on different days. (b) Graphical representation of wound healing in rats on different days.

**Figure:**
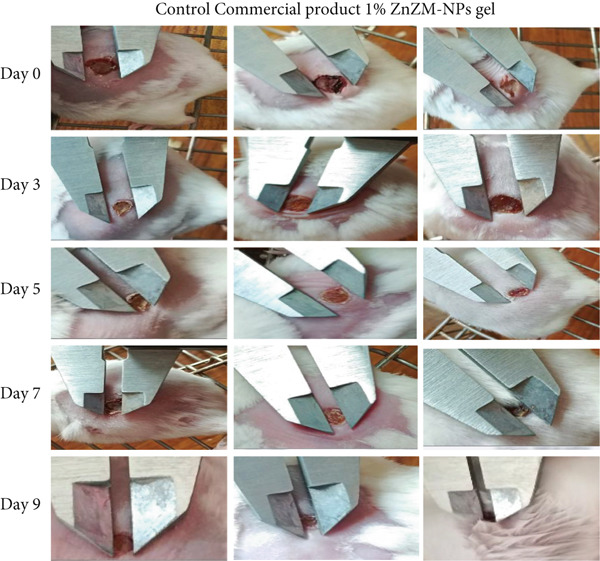
(a)

**Figure:**
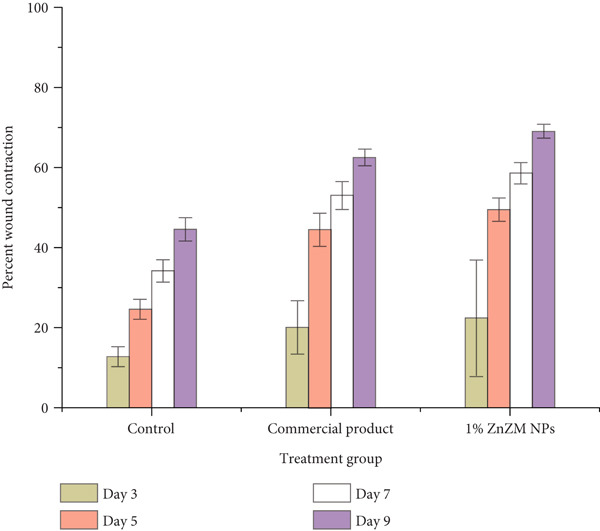
(b)

The wound contraction rates across all groups were statistically analyzed (Figure 5b). ANOVA revealed that both the topical gel‐treated group (Group 2) and the commercial product‐treated group (Group 3) exhibited significantly higher percentage wound contraction (*p* < 0.05) than the untreated control group. Consistent with previous studies, ZnO NP treatment substantially reduced the wound healing time [30]. Statistical comparison among treatment groups revealed that the ZnZM NP gel group demonstrated a significantly higher percentage of wound contraction (*p* < 0.05) than the commercial product on Days 5, 7, and 9. However, no statistically significant difference in wound healing was observed on Day 3.

### 3.5. Histopathological Examination

Microscopic examination of the skin tissue of the wounds was performed on Day 10. Figure 6 shows the dissected tissue samples from the three animal groups. In Group 1 (control, Figure 6a), slow wound healing was evident, with exposed uncovered wound surfaces, occasional fibroblasts, absence of macrophages, and no visible blood vessels. Group 2 (standard, Figure 6b) exhibited moderate wound healing, characterized by fewer fibroblasts, macrophages, and blood vessels. In contrast, Group 3 (1% ZnZM NP gel, Figure 6c) demonstrated near‐complete wound healing, with a significant reduction in the wound area, moderate presence of fibroblasts, macrophages and blood vessels, and normal regeneration of hair follicles. These histopathological findings align with those of previous studies conducted by Pammi, Padavala et al. and indicate the maximal wound‐healing ability of the ZnZM NP–loaded gel [23].

Figure 6Microscopic images of the wounded areas on Day 10 in different groups: (a) control, (b) standard, and (c) ZnO‐NP gel.

**Figure:**
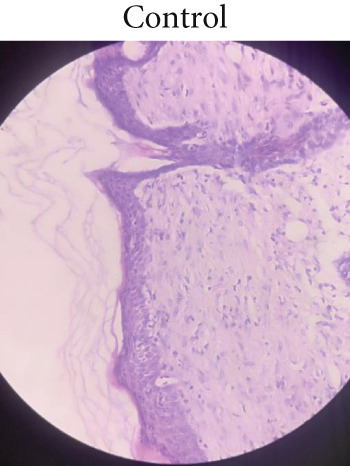
(a)

**Figure:**
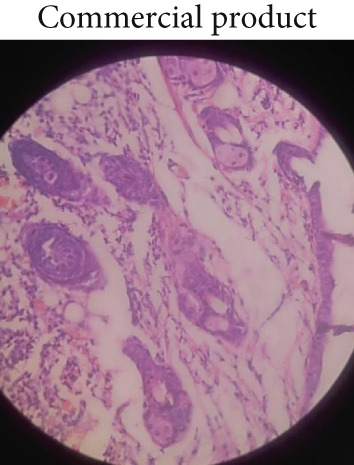
(b)

**Figure:**
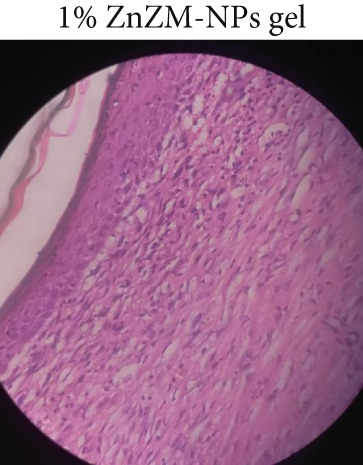
(c)

## 4. Conclusions

The present study demonstrated the successful green synthesis of zinc oxide nanoparticles (ZnZM NPs) using *Z*. *mays* leaf extract, confirming the plant′s phytochemicals as effective reducing and stabilizing agents. The synthesized nanoparticles exhibited distinct physicochemical features, including partial sphericity, crystalline structure, and nanoscale size distribution with an average crystallite size of 10.86 nm. FT‐IR analysis confirmed the involvement of bioactive compounds in nanoparticle formation and stabilization processes.

Biologically, ZnZM NPs exhibited antibacterial activity against *E. coli*, *P. aeruginosa*, and *K. pneumoniae*, demonstrating their potential to inhibit bacterial proliferation associated with wound infections. Moreover, the nanoparticles exhibited significant antioxidant activity, as evidenced by their Fe^3+^‐reducing and free radical‐scavenging abilities. These combined properties likely contributed to the observed enhancement in wound healing. Topical application of ZnZM NP–incorporated gel notably accelerated wound contraction and epithelialization in rats compared with the control and standard treatment groups. Histopathological analysis further confirmed superior collagen deposition, re‐epithelialization, and angiogenesis in treated wounds, suggesting that ZnZM NPs promote effective tissue regeneration.

Collectively, these findings indicate that ZnZM NPs possess multifunctional therapeutic potential as antibacterial, antioxidant, and wound‐healing agents. Their eco‐friendly synthesis route, cost‐effectiveness, and strong biological efficacy highlight their promise as sustainable nanomedicine for wound management and related biomedical applications.

### 4.1. Study Limitations

The present study was limited by its use of a small animal model and a restricted range of bacterial strains, which may not fully represent the clinical conditions. Long‐term toxicity, biodistribution, and biocompatibility assessments were not conducted. Future studies should focus on the detailed mechanisms, safety, and large‐scale validation to support the clinical translation of ZnZM NPs.

## Disclosure

All authors read and approved the final manuscript.

## Conflicts of Interest

The authors declare no conflicts of interest.

## Author Contributions

Aqsa Khalid: investigation, formal analysis, methodology, data curation. Raheela Waheed and Zermina Rashid: supervision, conceptualization, formal analysis, data interpretation, writing and editing of manuscript. Farah Deeba: writing—original draft preparation, review and editing. Ambreen Aleem: writing—review and editing, formal analysis, technical support. Mohamed Deifallah Yousif: writing—review and editing, validation, software, formal analysis.

## Funding

No funding was received for this manuscript.

## Supporting information


**Supporting Information** Additional supporting information can be found online in the Supporting Information section. Supporting data contain information about the preparation and evaluation of ZnZM NPs NPs–loaded carbopol gel (Figure S1).

## Data Availability

The data that support the findings of this study are available from the corresponding authors upon reasonable request.
