# Supplementary material for: Zea mays–Derived Zinc Oxide Nanoparticles Exhibiting Enhanced Antioxidant, Antibacterial, and Wound‐Healing Activities
Source: Biomed Res Int. 2026 Feb 4;2026:2670207. doi: 10.1155/bmri/2670207 (PMC12872593; doi:10.1155/bmri/2670207)
Supplement: Supplementary file 1 — Supporting Information Additional supporting information can be found online in the Supporting Information section. Supporting data contain information about the preparation and evaluation of ZnZM NPs NPs–loaded carbopol gel (Figure S1). [file BMRI-2026-2670207-s001.docx]

**Supplementary Materials**

**Preparation and evaluation of ZnZM-NPs loaded carbopol gel**

Carbopol gel was prepared as described in the methods section above (main document section 2.6). The resulting gel was homogeneous, with no clog or foreign particles. The physical appearance of blank carbopol gel and 1% ZnZM-NPs loaded carbopol gel is shown in Fig. S.


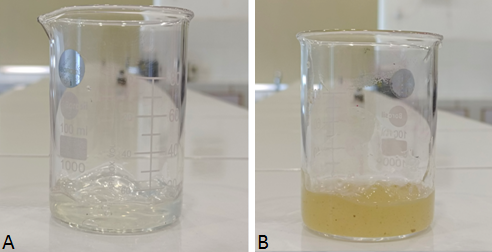


**Fig. S.** Formulations of carbopol gel. A is blank and B is ZnZM-NPs-loaded carbopol gel.

The Fig. S. showed that, the blank carbopol gel is transparent, while the 1% ZnZM-NP-loaded gel is brown. Both formulations were homogeneous in appearance with a pH in the range of 6.55-7.00, which is suitable for application on skin with less chances of skin irritation. The loaded gel showed no signs of erythema and/or edema upon application to the rat skin (skin irritation test), suggesting that the formulation is biocompatible and suitable for further evaluation in vivo, in wound healing studies.
